# Spatial patterns of West Nile virus distribution in the Volgograd region of Russia, a territory with long-existing foci

**DOI:** 10.1371/journal.pntd.0010145

**Published:** 2022-01-31

**Authors:** Natalia Shartova, Varvara Mironova, Svetlana Zelikhina, Fedor Korennoy, Mikhail Grishchenko

**Affiliations:** 1 Faculty of Geography, Lomonosov Moscow State University, Moscow, Russia; 2 FGBI Federal Center for Animal Health (FGBI ARRIAH), Vladimir, Russia; 3 Faculty of Geography and Geoinformatics, HSE University, Moscow, Russia; Fundacao Oswaldo Cruz, BRAZIL

## Abstract

Southern Russia remains affected by West Nile virus (WNV). In the current study, we identified the spatial determinants of WNV distribution in an area with endemic virus transmission, with special reference to the urban settings, by mapping probable points of human infection acquisition and points of virus detection in mosquitoes, ticks, birds, and mammals during 1999–2016. The suitability of thermal conditions for extrinsic virus replication was assessed based on the approach of degree-day summation and their changes were estimated by linear trend analysis. A generalized linear model was used to analyze the year-to-year variation of human cases *versus* thermal conditions. Environmental suitability was determined by ecological niche modelling using MaxEnt software. Human population density was used as an offset to correct for possible bias. Spatial analysis of virus detection in the environment showed significant contributions from surface temperature, altitude, and distance from water bodies. When indicators of location and mobility of the human population were included, the relative impact of factors changed, with roads becoming most important. When the points of probable human case infection were added, the percentage of leading factors changed only slightly. The urban environment significantly increased the epidemic potential of the territory and created quite favorable conditions for virus circulation. The private building sector with low-storey houses and garden plots located in the suburbs provided a connection between urban and rural transmission cycles.

## Author summary

West Nile fever is a zoonotic disease caused by flavivirus infection that dispersed circumglobally by the end of the 20th century. There is the long-existing endemic area in southern Russia (Volgograd region) that remains affected by West Nile virus (WNV) with the maximum number of reported cases in the country. The natural features of the region formed favorable conditions for long-term WNV circulation. At the same time, the urban environment could pose greater hazards for WNV infection in humans. Road density, distance from water bodies, land surface temperature and altitude were identified as the most significant spatial determinants of WNV distribution. The private building sector with low-storey houses and garden plots located in the city suburban area provided connections between urban and rural transmission cycles. Climate change also may favor WNV transmission as increased temperatures affect the sum of effective temperatures necessary for virus extrinsic replication. A statistically significant correlation between WNV human cases and the sum of effective temperatures was found during the last 20 years.

## Introduction

West Nile fever (WNF) is a zoonotic disease caused by infection with a flavivirus (*Flaviviridae* family) that is endemic in Africa, the Middle East, and South Asia, and then dispersed circumglobally by the end of the 20th century [1,2]. Viral transmission originally occurred between mosquitoes and birds, but currently, circulation has involved more hosts and vectors outside the original ecological niche [3]. Transmission *via* mosquitoes may cause severe infection in humans and horses that are dead-end hosts for the virus.

Since the middle of the 20th century, there has been evidence of a changing geographical distribution of West Nile virus (WNV), as well as a shift in epidemiology and activity of the virus. The emergence of WNF in new regions has been associated with climate change, however transmission, distribution, and incidence of West Nile virus disease are influenced by multiple factors [4]. More information about WNV spatial distribution can be found in worldwide reviews [5–9] and regional studies in Europe [10–13], Africa and Eastern Mediterranean region [14–15] and North America [16–18].

### West Nile virus spatio-temporal distribution in Russia

**Fig 1:**
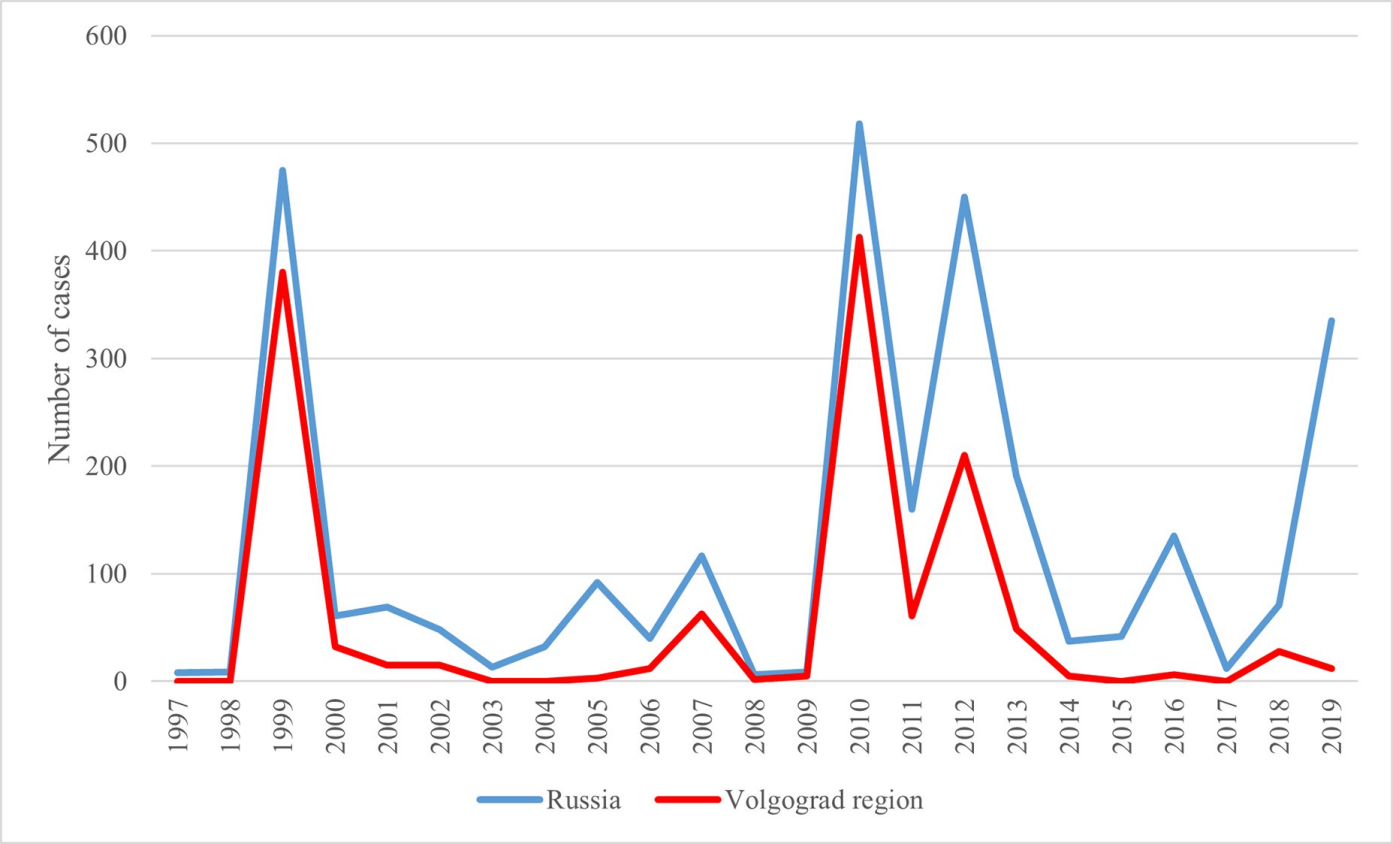
WNV laboratory confirmed cases among patients with fever or neuroinvasive disease in Russia and the Volgograd region, 1997–2019. Source: official records of Federal Service for Surveillance on Consumer Rights Protection and Human Wellbeing (Rospotrebnadzor) upon request.

**Fig 2:**
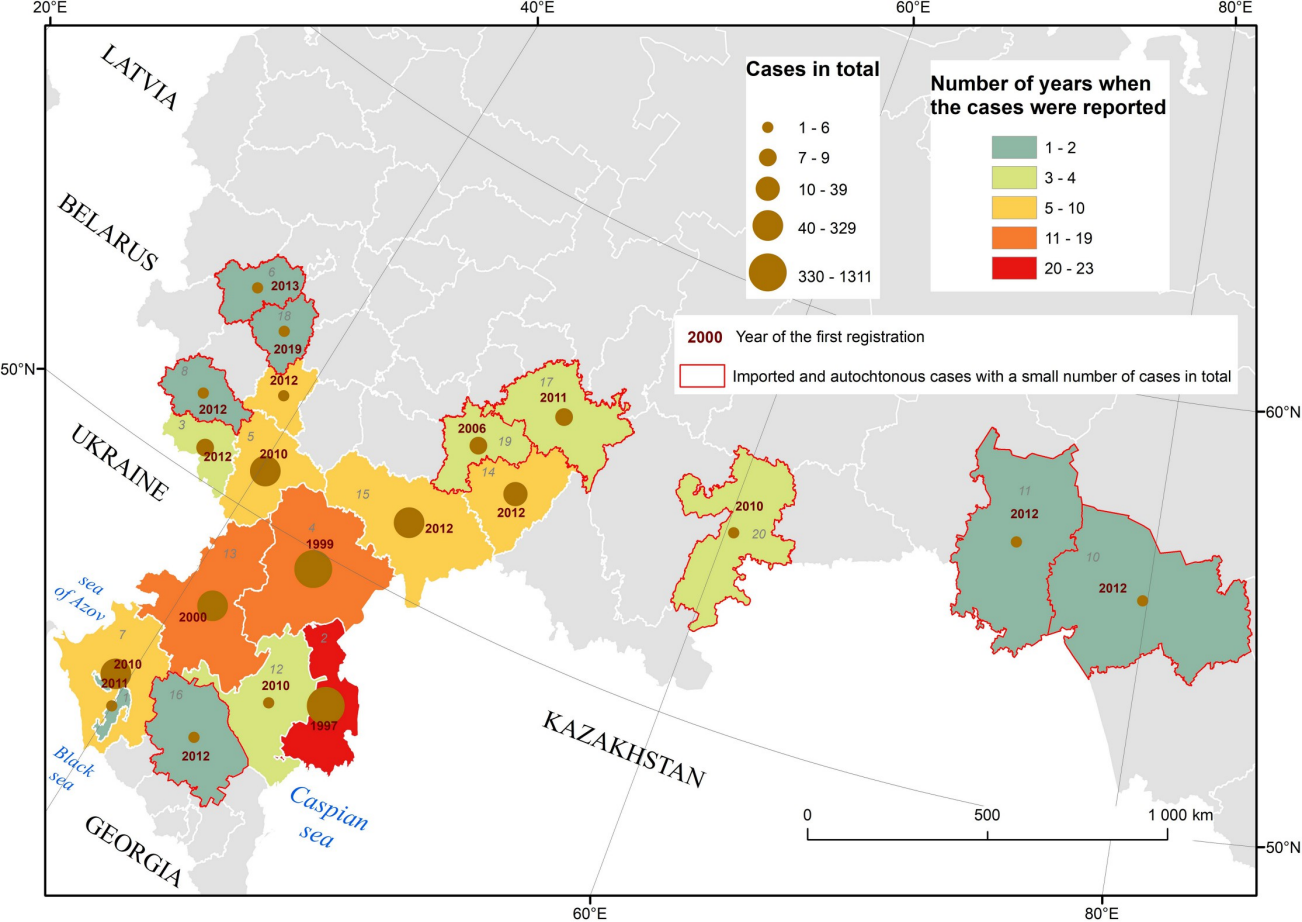
Spatial distribution of WNV laboratory confirmed cases among patients with fever or neuroinvasive disease per administrative units in Russia, 1997–2019. Source: official records of Federal Service for Surveillance on Consumer Rights Protection and Human Wellbeing (Rospotrebnadzor) upon request. Contains information from OpenStreetMap and OpenStreetMap Foundation, which is made available under the Open Database License. *The numbers indicate*: *1 Adygea; 2 Astrakhan region; 3 Belgorod region; 4 Volgograd region; 5 Voronezh region; 6 Kaluga region; 7 Krasnodar krai; 8 Kursk region; 9 Lipetsk region; 10 Novosibirsk region; 11 Omsk region; 12 Kalmykia; 13 Rostov region; 14 Samara region; 15 Saratov region; 16 Stavropol krai; 17 Tatarstan; 18 Tula region; 19 Ulyanovsk region; 20 Chelyabinsk region*.

In Russia, WNV was first isolated from larvae and nymphs of *Hyalomma marginatum (syn*. *H*. *plumbeum)* ticks in the Astrakhan region (hereinafter, the ‘region’ refers to an official territorial administrative unit) in the Volga River Delta in 1963 and was associated with Lineage 1 [19,20]. The first laboratory confirmed WNF cases were detected in the Astrakhan region in 1967 [21]. However, the first official WNF case was not recorded from the same region until 1997. The first documented outbreak occurred in 1999 in the Astrakhan and neighbouring Volgograd regions (475 cases) and again WNV Lineage 1 was detected [20]. The most extensive outbreak occurred in 2010, with 521 cases (Fig 1) [22]. The virus spread to seven regions, with the Volgograd region most affected (413 cases). Additional human cases later were reported from other southern regions of Russia, in addition to the long-existing foci in the Astrakhan and Volgograd regions [23] (Fig 2). However, the Volgograd region has remained the area with the maximum number of reported cases. The second major outbreak in 2010 was associated with Lineage 2 [24]. Sequencing of virus strains recently isolated from European Russia showed they were different from the strains isolated 20–30 years ago [25]. The present strains are similar to the strains from Europe, identificated as Lineage 2 [24].

### Hosts and vectors

In the natural environment, the transmission cycle involves wild birds as principal hosts and mosquitoes that feed on birds as vectors [26]. Fifty-six bird species have been involved in virus transmission in wetlands and surroundings of the Volga delta [27]. Storks and other Ciconiiformes were most frequently identified as a virus host among wild birds as well as the great cormorant (*Phalacrocorax carbo*), Eurasian coot (*Fulica atra*), common moorhen (*Gallinula chloropus*), great crested grebe (*Podiceps cristatus*), and seagull and sterna (family Laridae) (based on virus detection among wild birds) [27,28]. Synanthropic birds, primarily rooks, crows, pigeons, and sparrows, have been involved in the transmission cycle in urban areas [29,30].

There are fifteen mosquito species, mainly of the genus *Culex* and, less commonly, of *Aedes* and *Anopheles* genera, that have been considered potential vectors of WNV in Russia [31]. *Culex* spp. mosquitoes (*Culex pipiens L*. and *Culex modestus Fic*.) are most epidemiologically important [32–34] and have been confirmed to be competent to transmit WNV [35]. *Culex pipiens* is the main vector within bird populations, whereas *Cx*. *modestus* dominates in reeds near their larval habitat as well as in urban habitats and can be a bridge vector from birds to humans. The autogenous anthropophilic biotype *Cx*. *pipiens molestus* has been found in flooded basements and may be involved in urban settings [36].

Ticks from Ixodidae (mainly *Hyalomma marginatum*) and Argasidae (mainly *Ornithodoros coniceps*) families have been implicated as having a minor role in transmission [2], with their important role in preserving the virus during winter. The infection rate of all tick metamorphosis phases is much higher compared to mosquitoes [37,38].

### Environmental drivers

The transmission of WNV depends on several environmental factors. Ambient temperature is a direct factor that influences viral replication and growth rates of mosquitoes [26]. Virus transmission by vectors is possible provided that a certain amount of heat (effective temperature or degree days) is accumulated. Degree days are calculated by summing the daily excess of the average daily temperature above a certain threshold value [39]. A total of 109-degree days above the threshold of 14.3°C seems necessary for WNV replication [40]. An increase in temperature reduces the length of the mosquito gonotrophic cycle and shortens the extrinsic incubation period of the virus in the vector [40–42]. However, some studies showed that increased temperature can reduce adult mosquito longevity [43–45]. According to experiments [43], *Cx*. *pipiens f*. *pipiens* longevity decreased with increasing temperature and did not exceed 12 days at 30°C. The maximum mosquito survival was about 130 days at 15°C and the optimal temperatures for the co-occurrence of virus replication and *Cx*. *pipiens* can range from 14.3 to 30°C [44,45]. However, shortening of the extrinsic incubation period due to warm temperatures can compensate for the reduced survival of mosquitoes, making transmission more efficient. For example, transmission rates of northern European *Cx*. *pipiens* increased from 0% to 33% in the temperature range from 18°C to 28°C [35].

Precipitation can affect mosquitoes and WNV transmission in different ways. On the one hand, precipitation may increase the WNF incidence due to a higher abundance of mosquitoes [26,46] and their ability to survive at a higher humidity. Conversely heavy rainfall can ‘flush out’ larvae from their habitat [47]. Weak correlations and inconsistent patterns between precipitation and *Cx*. *pipiens* dynamics were found across Europe [48]. Such a weak correlation may be due to the inconsistent nature of precipitation and also due to the fact that the natural and urban environments react differently to rainfall. Although for wetlands, it is important to consider the accumulation of precipitation in periods preceding adult mosquito sampling, for urban environments this factor does not have much effect as most precipitation is lost from impervious surfaces into drainage systems. In urban environments it may matter in case of light rainfall or landscape irrigation that can improve mosquito production.

The distribution of mosquito habitat is determined by vegetation cover and the presence of water bodies in the natural environment [49,50]. Wetlands are the most important ecosystems for enzootic transmission due to the presence of resident and migratory bird and mosquito populations, particularly *Cx*. *pipiens* [51]. Urban areas with a simple avian fauna consisting of high density of competent hosts can provide even more efficient transmission. Indices such as the normalized difference vegetation index (NDVI) and normalized difference water index (NDWI) have been identified as risk indicators for WNV outbreaks in previous studies in Europe [52], North America [53], and Russia [54]. In cities, conditions often favor *Cx*. *pipiens* production as they utilize stagnant and polluted water bodies, such as drainage ditches, ponds, abandoned barrels, and other man-made reservoirs, as well as flooded basements of buildings [55,56].

The role of elevation in WNV transmission depends on the ecology of the mosquito vectors involved. Usually, the abundance and mosquito species diversity are the greatest in lowlands [26,57]. Often, the risk of infection decreases with increasing elevation [57–60].

WNV transmission can occur in both rural and urban environments. The urban environment can contribute to WNV transmission by involving synanthropic birds and creating additional man-made mosquito breeding sites [31,61–63]. Johnson et al. [64] showed that residential areas contained significantly higher proportions of WNV-competent mosquito species and avian host species when compared to adjacent urban wetlands. Urban environment heterogeneity may cause decelerating speed of pathogen spread waves in incipient epidemics [65]. Overall, the urban environment influences the distribution and mobility of the human population and other dimensions of social life.

In this paper, we aimed to identify the spatial determinants of WNV distribution in an endemic area in southern Russia focusing on the urban environment and the assessment of thermal suitability for WNV transmission. We analyzed the contribution of various environmental and urban variables as determinants of WNV distribution according to different scenarios of spatial modelling. Then, we calculated the effective temperatures and length of periods during the years when replication and infection were possible. Finally, we explored trends in weather variables during recent years in association with climate change.

## Methods

### Study area

The study area included Volgograd city and its suburbs in southern Russia between 48.38° N and 49.07° N and 43.97° E and 45.11° E (Fig 2). This territory consisted of typical multi-storey urban areas as well as traditional villages and groups of summer cottages that belong to city dwellers and are used as summer vacation spots and for small-scale production of fruit and vegetables for personal consumption.

This area has a hot summer continental climate (Dfa) according to the Köppen climate classification [66]. The average temperature for the warmest month (July) is 23.6°C, and the average temperature for the coolest month (February) is -6.6°C (S1 Fig). The average annual precipitation is 348 mm, with the maximum in May and the minimum in February, April, and September [67].

**Fig 3:**
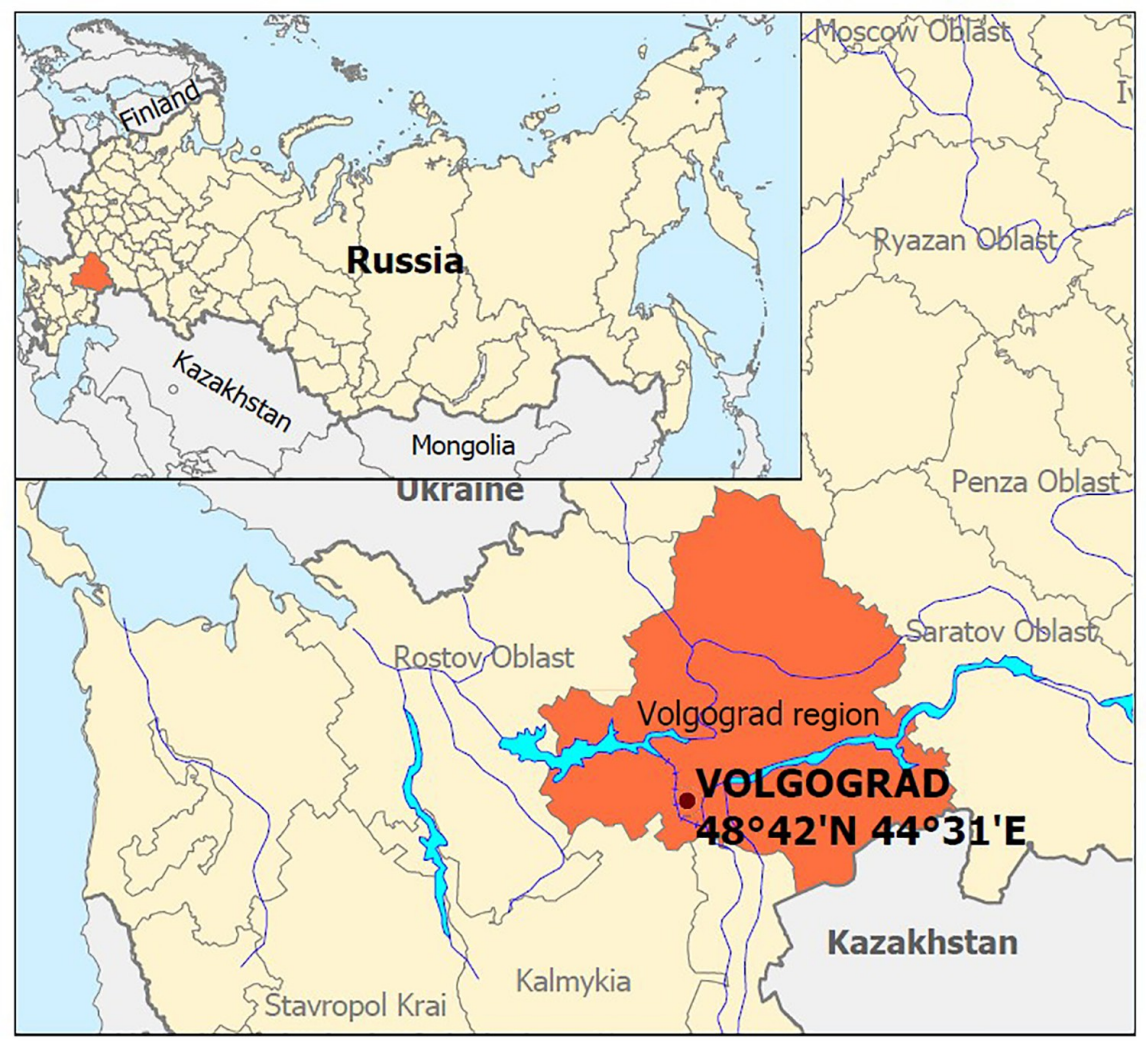
Study area–Volgograd city and its suburbs in southern Russia. Contains information from OpenStreetMap and OpenStreetMap Foundation, which is made available under the Open Database License.

Volgograd is a large industrial center located on the Volga River, with nearly one million inhabitants (Fig 3). The city stretches along the river for approximately 60 km. The immediate vicinity of the city on the right bank of Volga (to the West) is elevated arid steppes. In contrast, the immediate vicinity of the city on the left bank (to the East) is the Volga-Akhtuba floodplain.

Within the city, along with industrial zones and areas of urban development, there are lowlands with wetlands, flooded Volga islands with various rural settlements, and recreational zones. These islands provide a natural buffer between the large industrial city and floodplain ecosystems of the Volga-Akhtuba floodplain [68]. In the city territory, there are several small rivers with hydrophilous vegetation along the banks, many ponds, and a ravine and gully network as well as artificial water bodies of various size. The wetlands in the vicinity of Volgograd are crossed by many ancient river channels, lakes, and bogs and are part of the migratory routes of birds. Thus, in Volgograd, both rural and urban circulation of the virus can be maintained largely due to the diversity of mosquito habitats.

### Virus detection data

Data on virus detection in the environment and points of probable human infection were used to determine WNV distribution. The source of the data was the official record of the Federal Service for Surveillance on Consumer Rights Protection and Human Wellbeing (Rospotrebnadzor). Virus detection in the environment was carried out by epidemiologists of Rospotrebnadzor according to the methodological guidelines “MU 3.1.3.2600–10 Activities against West Nile fever in the Russian Federation” and included testing for virus by RT-PCR. Samples for testing included arthropods (ticks and mosquitoes), animal carcasses, and bird feces. Samples were collected from different places in natural and urban environments during 1999–2016. All locations positive for WNV RNA were recorded as virus detection sites.

A total of 29 places with a positive RNA test were found. Additional information about possible points of laboratory confirmed human infection during 2011 was added to strengthen the spatial analysis. Each human disease case was investigated by epidemiologists of Rospotrebnadzor to determine the probable point of infection. 2011 was considered a “typical” year, with an average number of officially reported WNF cases in Volgograd city and its neighbouring areas (Table 1). We assumed that these 55 points of infection corresponded to the most favorable places for WNV presence in the environment.

**Table 1:** The statistical analysis of human WNV cases in the Volgograd region, 1997–2019.

Minimum	Maximum	Mean	Std. deviation	Median	Cases 2011
0	413	57	116,205	12	61

**Fig 4:**
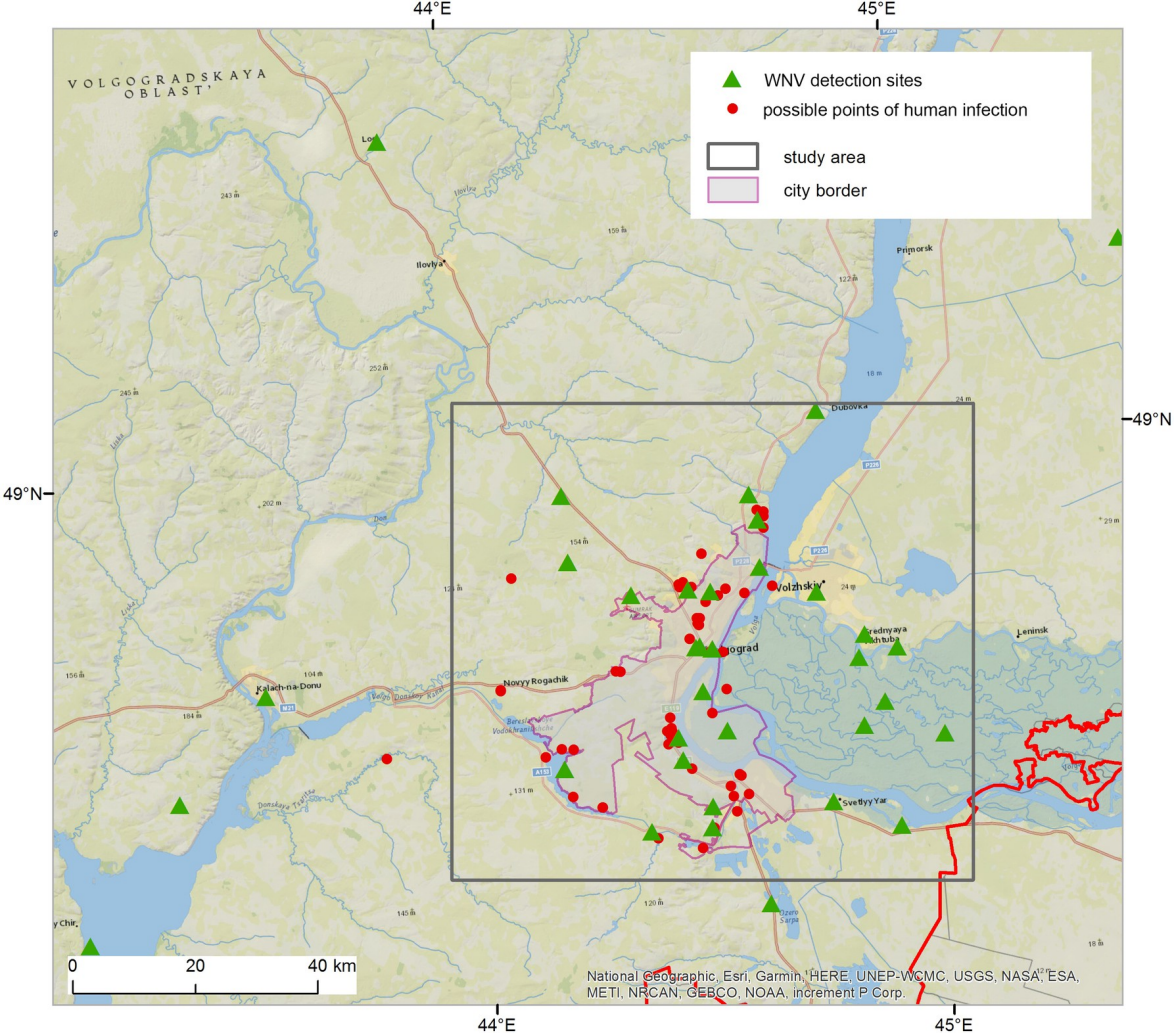
WNV detection sites (1996–2016) and possible places of human infection (2011) in the environment in Volgograd and neighbouring areas. Contains information from OpenStreetMap and OpenStreetMap Foundation, which is made available under the Open Database License.

Thus, we used a general dataset on WNV distribution in the environment, whether it was actual detection or potential presence. All 89 sites were geocoded and mapped using ArcGIS software (Fig 4).

### Environmental data

The daily data from the Volgograd weather station were used to analyze the effects of temporal change in temperature, precipitation, and relative humidity on the occurrence of human WNV cases during 1997 to 2019. This station is operated under the standards of World Meteorological Organization (WMO, ID-34561). Raw data were downloaded from the Russian Institute for Hydrometeorological Information—World Data Center (RIHMI-WDC, source: www.meteo.ru).

To focus on the spatial heterogeneity of environmental conditions, we examined the following environmental variables as natural environment predictors of WNV distribution: land surface temperature (LST), normalized difference vegetation index (NDVI), normalized difference water index by McFeeters (NDWI), distance from water bodies, and elevation. LST was calculated through Landsat thermal infrared imagery converting Band 6 spectral radiance to the brightness temperature according to the “Landsat 7 (L7) Data Users Handbook—Version 2.0” (https://www.usgs.gov/media/files/landsat-7-data-users-handbook) and then upgraded by making atmospheric correction using MODTRAN model [69] considering land surface emissivity based on previous methods [69–71]. Cloud screening and handling the resulting missing data was carried out in a semi-automatic mode: for each image, a threshold value for the brightness of pixels that correspond to clouds was determined and all pixels with values less than the threshold were excluded from further analysis. LST was used in spatial modelling as a proxy for air temperature, taking into account the difference between air temperature and LST in areas with low vegetation [72–74]. NDVI [75] was calculated using red and near-infrared wavelengths. NDWI [76] was determined using green and near-infrared wavelengths. For each of the three variables, the average values for all images were calculated using a raster calculator of ArcGis software.

The urban environment was represented by three variables: building density, road density, and railway density. The inclusion of the railway density was included to take into account frequent summer trips of city dwellers out of the city to personal household plots (gardens) for summer vacations or weekends. Such seasonal migrations are very typical for the urban population of Russia and railways are the main transport mode for these purposes. In this case, ‘city dwellers’ moving to suburban potential places of infection is possible. Variables in the urban environment were based on vector data from OpenStreetMap and were calculated in ArcGis software. The density of linear objects was calculated using the Kernel algorithm, the building density was calculated as the ratio of the area of buildings to the total area on a regular grid cell.

Population density was used as a bias factor to account for the assumption of greater likelihood of WNV detection in places with a higher population density (Table 2). A population density raster (S2 Fig) was created within the study area and incorporated as a bias file into the model. Maxent software factors out the bias by assigning weights to the random background data during modeling [77].

**Table 2:** Response, explanatory, and offset variables for spatial modelling of heterogeneity of environmental conditions for WNV distribution.

		Variable	Raw data source	Spatial / time resolution	Reference
**Response variables**	WNV localization in the environment	Localization of virus detection in the environment	Official Rospotrebnadzor records	1999–2016	**-**
		Localization of possible human infection		2011	**-**
**Explanatory variables**	Natural environment–temperature, vegetation cover, presence of water bodies and topography	LST	Landsat 7 & Landsat 8 https://earthexplorer.usgs.gov/.	Images in the thermal spectrum have 60 m (Landsat 7) and 100 m (Landsat 8) cell size. Rasters are resampled to 30 m cell size. Series of satellite images included 21 May– 7 September 2010 & 26 May– 2 September 2011 (19 days)	[53,72]
		NDVI			[52,54]
		NDWI			[78]
		Distance from the water bodies	Open Street Map contributors 2020 https://www.openstreetmap.org/	30 m	[78]
		Elevation	Digital model ALOS DEM https://www.eorc.jaxa.jp/ALOS/en/aw3d30/index.htm	30 m	[56]
	Urban environment—–built-up environment and population mobility	Building density	Open Street Map contributors 2020 https://www.openstreetmap.org/	60 m	[56]
		Road density			[79]
		Railway density			
**Offset variables**		Population density	Global High Resolution Population Denominators Project https://www.worldpop.org/geodata/summary?id=49725	30 m	[80]

### Spatial modelling of environmental suitability for WNV transmission

The environmental suitability for WNV was modelled using an ecological niche modelling (ENM) approach using MaxEnt software. Maxent is a machine learning algorithm based on the maximum entropy approach [81]. This method is widely used to determine the habitat suitability for a particular species according to the environmental conditions [82] or to model the suitability of an area for disease occurrence considering limiting and risk factors [83]. This maximum entropy technique is based on the principle of geospatial regression. It describes the known distribution of the study phenomenon using presence-only data [81,84]. The program randomly generates absence points of the phenomenon and takes a set of environmental predictors across a user-defined landscape that is divided into grid cells [85]. As a result, this method produces a map, where each grid cell expresses the suitability as a function of the environmental variables, based on the input presence data.

In the present study 89 sites of WNV detection (29 sites of virus detection and 55 probable human infection sites) in the environment were used as presence data, while environmental data were presented by natural and/or urban environmental variables (Table 2). According to recommendations found elsewhere [86,87], the number of input presence locations was sufficient for accurate model development. According to these studies the minimum limit sample size varied around 15–25 presence observation with the optimum size no less than 30 [86,87]. Despite the presence of unstable results when applying models over a large area, the use of the low sample size in smaller radius around the occurrence points could provide good working models [88]. In contrary to widespread non-parametric tests, such as generalized linear mixed models or generalized additive mixed models, that usually require numerically powerful sampling, MaxEnt can be used specifically for small sample sizes [86–89].

Prior to the MaxEnt analysis, the environmental variables were tested for multicollinearity using the ’usdm’ package in the R 4.0.3 software environment [90]. It was revealed that the only pair of correlated variables was the NDVI and NDWI (Pearson correlation coefficient above 0.85), of which the former had a larger variance inflation factor (VIF). According to VIF values NDVI was excluded from the further analysis. However, we checked separately the contribution of NDVI and NDWI in models and results were similar (S1 Table). Three simulation scenarios were developed (Table 3).

**Table 3:** The set of environmental predictors in different combinations for modelling.

	Input presence data		Environmental variable sets		
	Possible human infection sites	Sites of virus detection		Environment	Urban environment
Model 1		✓		✓	
Model 2		✓		✓	✓
Model 3	✓	✓		✓	✓

For the model parameterization, we chose the default regularization multiplier of 1 with 10,000 background points (S2 Table). Bootstrap validation was chosen with 100 replications in which a maximum of 5,000 iterations were allowed for training to reach a 0.00001 convergence threshold. The mean values and confidence interval limits of the outputs were reported and presented as diagrams. Assessment of the contribution of each variable was made by the ‘jack-knife’ method based on a comparison of model performance with (1) the sequential omitting of each variable and (2) modelling with only one variable. The accuracy of the models was estimated by the area under the curve indicator (AUC), which assesses the ability of the model to correctly distinguish between true presence and pseudoabsence data, with AUC>0.7 considered an acceptable value [91].

### Statistical analysis of thermal suitability for WNV transmission

Warming temperature and other climatic parameters connected with climate change should be taken into account when evaluating the risk of WNV disease spread [4]. Since the 1990s, both global and regional trends in climate change that may affect the distribution of vector-borne diseases have been observed in Russia [92]. Therefore, we analyzed the change in meteorological conditions that determine the favorability for WNV transmission during the period of official registration of human cases (1997–2019).

First, the suitability of thermal conditions for WNV transmission was assessed based on the accumulation of 109 degree-days (or sum of the effective temperatures, ETs, ^0^C) which is necessary for the completion of virus extrinsic replication [40]. The calculation of daily degree days was based on a modified sinusoidal method [93,94]. The duration of the season of effective temperatures (SETs) and season of effective infectiveness of mosquitoes (SEI) in days were calculated in addition to the ET. SET is the period during which the average daily temperatures consistently remained above the threshold of 14.3°C, which corresponds to the warm period of the year (usually from March to October in Volgograd). SEI is the period during which viral replication in mosquitoes is possible and mosquitoes can infect humans, mammals, and birds. The duration of SEI depended on how quickly the effective temperatures were accumulated and how long the period with daily temperatures above 14.3°C lasted. This period starts at the same moment as SET but stops earlier due to a shorter time during which there is favorable temperature for full viral replication at the end of the warm season (the infection of mosquitoes is not effective in this case). Before the end of SET, the necessary amount of heat required for the virus replication must be accumulated. The SEI end date is the date after which this amount of heat does not accumulate and effective mosquito infection is not possible.

Second, to evaluate the contemporary changes in the meteorological conditions, we performed a linear trend analysis of ET, SET, SEI, as well as daily temperature, precipitation, and relative humidity for the period of official registration of the disease from 1997 to 2019.

The Mann-Kendall nonparametric statistical test was used to evaluate the significance level of the trends, and Sen’s slope estimator [95] was used to evaluate the rates of changes. These statistical methods can be applied even if the time series do not conform to a normal distribution [95,96]. Finally, Sen’s slope coefficients (k) and minimum confidence levels (p) at which the trends would be statistically significant according to the Mann-Kendall test were obtained for each analyzed parameter.

Third, we analyzed the correlation between human WNV cases during 1997–2019 in Volgograd region and variables of thermal conditions for WNV transmission. The Kendall rank correlation coefficient was used to identify the correlation and generalized linear negative binomial regression model was used to analyze the year-to-year variation.

Statistical analysis was performed in R software using the external packages “trend”, “modifiedmk”, and “MASS”.

## Results

### Environmental suitability assessment

As a result of the modelling of WNV distribution, environmentally suitable areas were identified. For each scenario under consideration, a model was obtained that was characterized by sufficiently high quality with the AUC ranging from 0.86 to 0.93 (Table 4).

**Table 4:** Variable contributions according to various spatial models.

Variables	Contribution %		
	Model 1	Model 2	Model 3
	Virus detection sites in the environment as presence data, natural environmental variables	Virus detection sites in the environment as presence data, natural and urban environmental variables	Possible human infection and virus detection sites in the environment as presence data, natural and urban environmental variables
LST	39.7	7.6	17.6
NDWI	14.1	6.1	3.9
Distance from the water bodies	23.8	12.5	7.7
Elevation	22.3	9.6	1.9
Building density	-	7.9	5.7
Road density	-	54.0	58.7
Railway density	-	2.4	4.4
**Model AUC**	**0.857±0.027**	**0.930±0.015**	**0.947±0.008**

The analysis of virus detection sites in the environment (Model 1) showed a significant contribution of surface temperature (39.7%), distance from the water bodies (23.8%), and elevation (22.3%) (Table 4). The contribution of water bodies identified by the NDWI was the smallest (14.1%) in this model.

When variables of urban environment were included (Model 2), the relative impact of factors changed. The density of roads was ranked first with a value of 54%. The distance from the water bodies remained important (12.5%) and it was the leading factor among all natural environmental drivers. The contribution of surface temperature and elevation was reduced significantly to 7.6 and 9.6%, respectively. The characteristics determined by NDWI, building and railway density did not make significant contributions to the model (with values of 6.1, 7.9, and 1.4%, respectively).

When the places of probable human infection sites were added to the sites of virus detection in the environment (Model 3), the road density remained the main factor, although its percentage increased slightly to 58.7%. The contribution of surface temperature increased to 17.6% and it was the second leading factor. The third factor was the distance from the water bodies (7.7%). The contributions of other factors were less than 5%.

**Fig 5:**
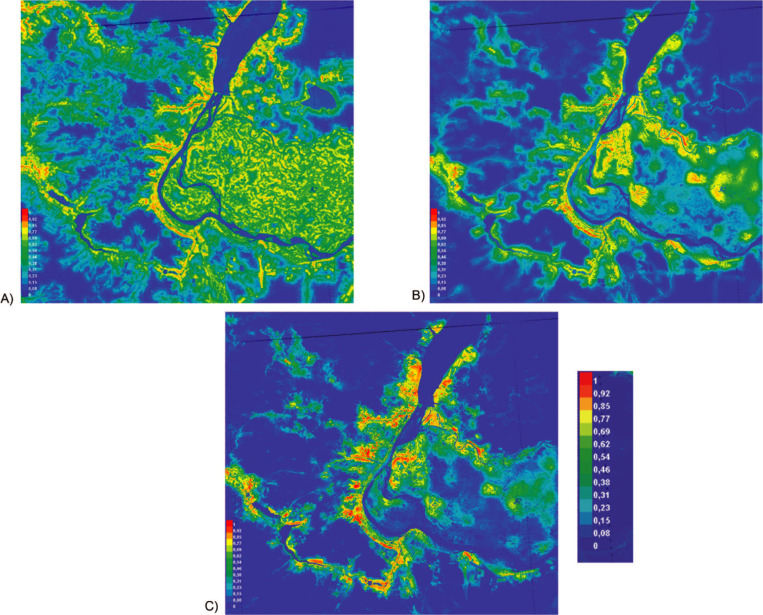
Environmental suitability for WNV distribution. (A) Model 1 based on the data on virus detection sites in the environment as presence data with natural environmental explanatory variables, (B) Model 2 based on the virus detection sites in the environment with natural and urban environmental explanatory variables, and (C) Model 3 based on the data on possible human infection and virus detection sites in the environment with natural and urban environmental explanatory variables. The colour indicates the degree of suitability.

Model 1 showed that the entire territory of the Volga-Akhtuba floodplain is suitable for WNV transmission (Fig 5A). The same suitability was observed along the Volga and Don rivers and their tributaries. An increase of suitability was identified in the immediate vicinity of water bodies.

The inclusion of urban environment variables in the modelling generally traced the same trends (Fig 5B). Visually, the suitability of areas near water bodies outside Volgograd seemed important in virus transmission. With the inclusion of probable human infection sites, the general tendency of confinement to water bodies remained the same (Fig 5C). At the same time, local areas with highest suitability were allocated to outside the central and the most populated part of the Volgograd city.

**Fig 6:**
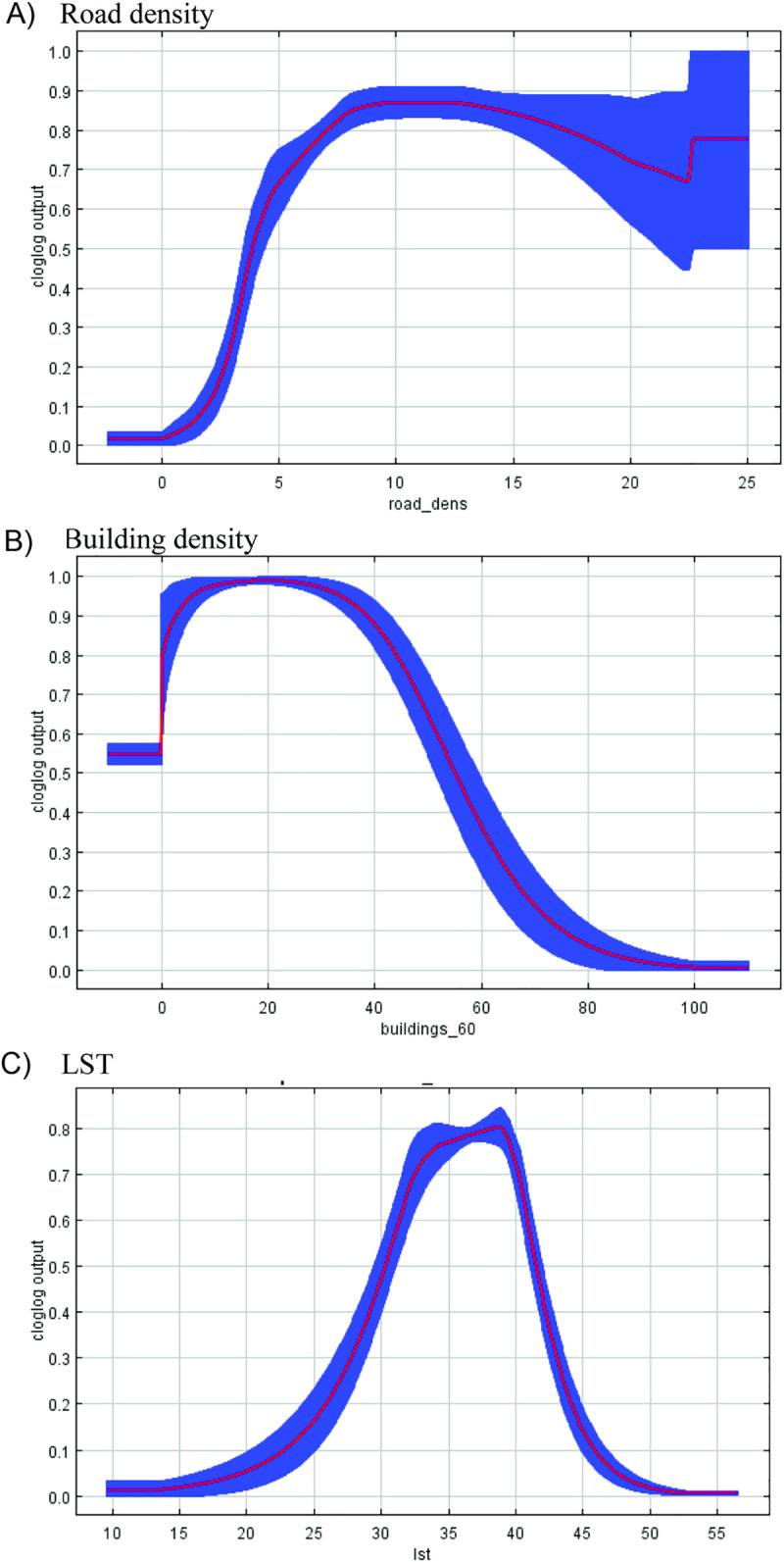
Response curves reflecting the influence of road density (A), building density (B) and LST (C) on the likelihood of the appearance of WNV distribution. The blue area shows the statistical significance of the response curve.

**Fig 7:**
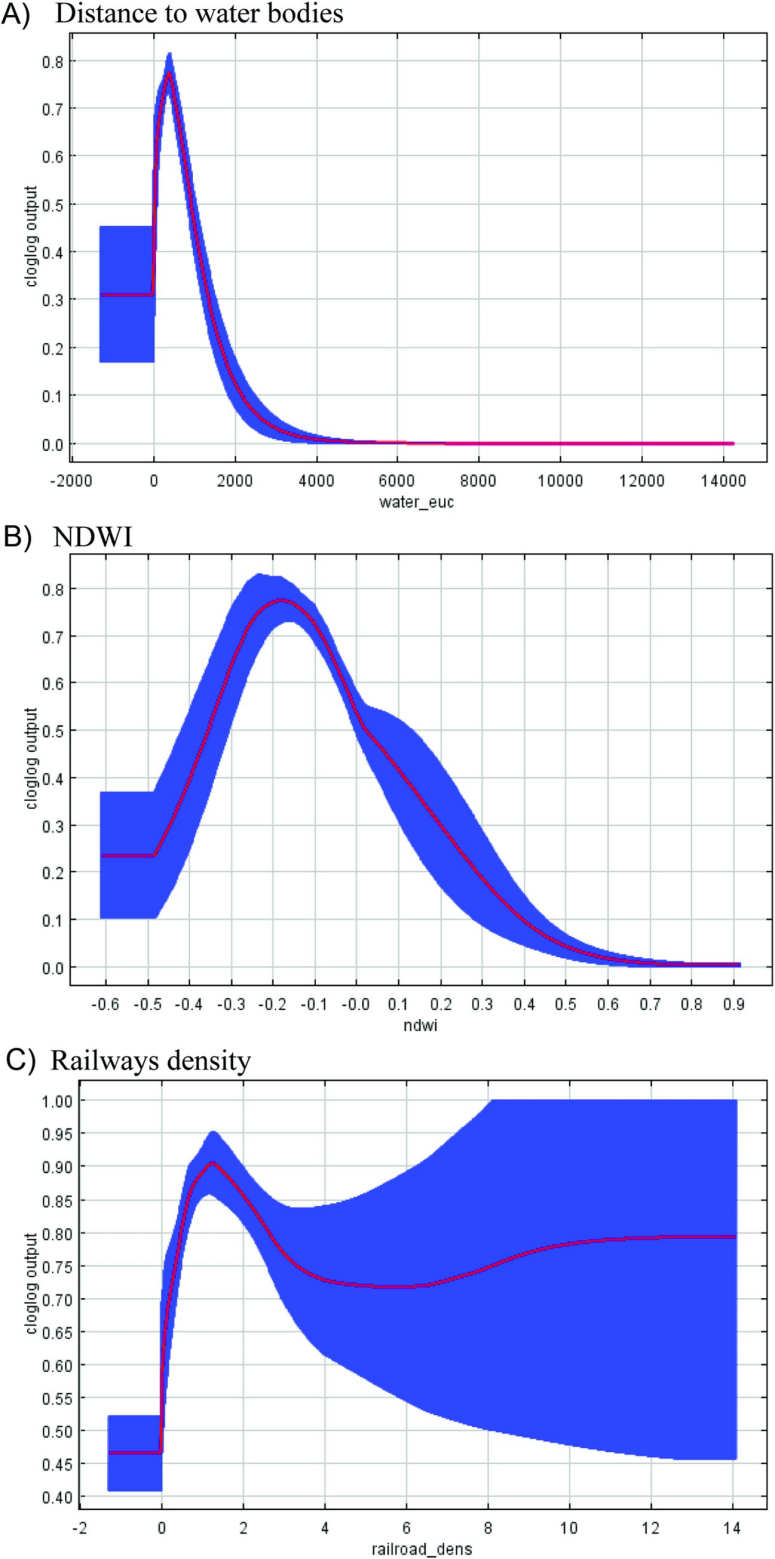
Response curves reflecting the influence of distance to the water bodies (A), NDWI (B) and railway density (C) on the likelihood of the appearance of WNV distribution. The blue area shows the statistical significance of the response curve.

**Fig 8:**
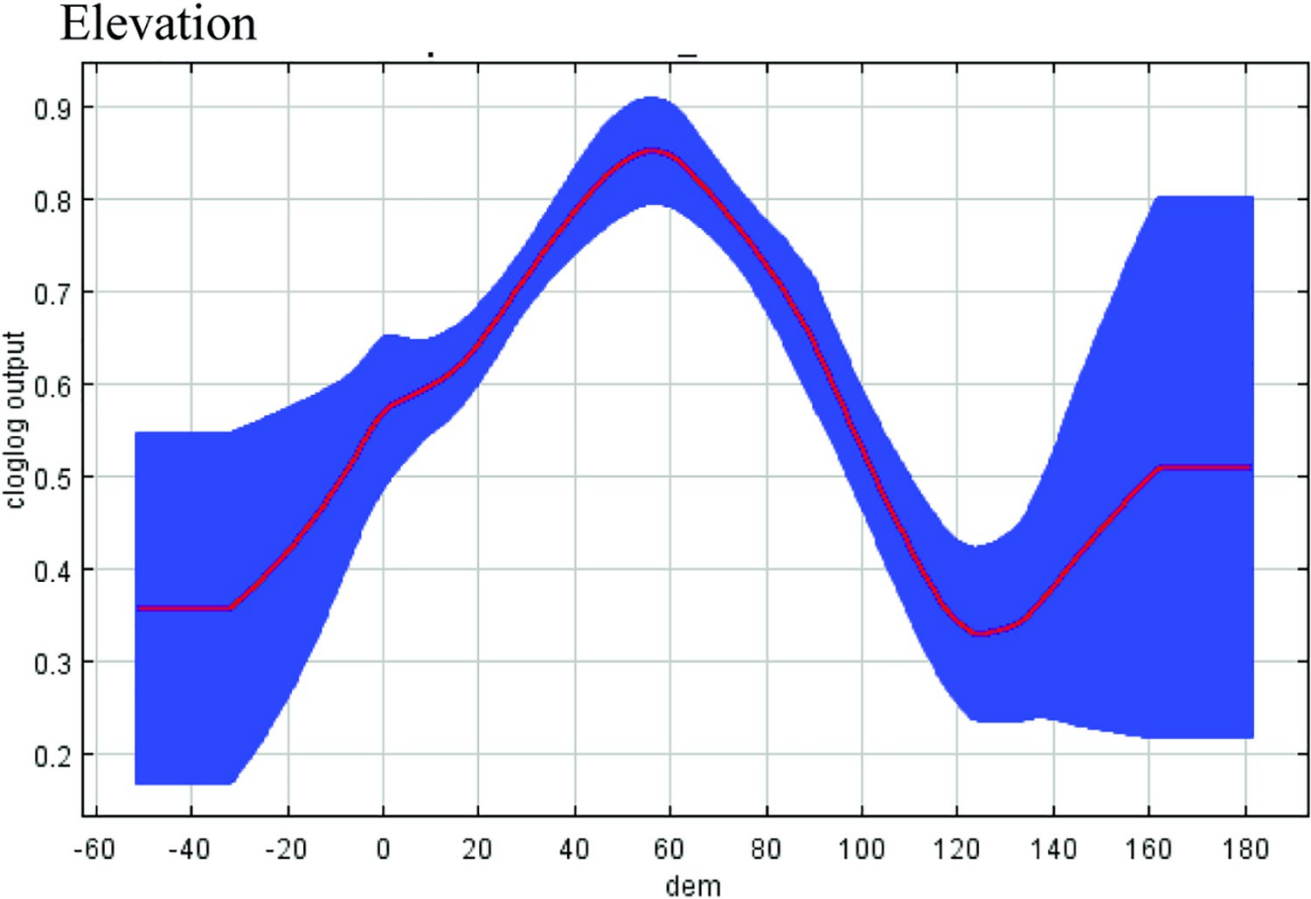
Response curves reflecting the influence of elevation on the likelihood of the appearance of WNV distribution. The blue area shows the statistical significance of the response curve.

Response curves defined the characteristics of each environmental metric (Figs 6–8). The highest probability of WNV occurrence was positively correlated with road density (Fig 6A). Increasing road density beyond a certain threshold produced an insignificant increase in transmission risk. In contrast, there was a high probability response to building density up to 40 sq. m. within a cell, with a subsequent drop in the response with a higher building density typical of industrial zones (Fig 6B). Optimum surface temperatures corresponded to approximately 33–40°C, which is typical for areas of residential urban development (Fig 6C). It should be noted that the surface temperature in the city is lower than that in the surrounding steppe areas (S3 Fig). The likelihood of virus detection was the greatest near water bodies at a distance of approximately 100 m and in areas with lower elevation of 50–60 m (Figs 7A and 8). The values of the NDWI showed the optimum in areas with herbaceous vegetation (Figs 7B and S4).

### Assessment of thermal suitability for WNV transmission

The average values of variables used to assess the suitability of temperature conditions for WNV transmission are presented in Table 5.

**Table 5:** Descriptive statistics and trend values for variables of thermal suitability and daily meteorological variables for WNV transmission between March and October from 1997 to 2019.

Variable	Minimum	Maximum	Mean	Std. deviation	Median	tau	Sen’s slope coefficient	p-value
Daily temperature (°C)	-9.10	40.00	18.13	8.08	17.1	0.05	0.0004	<0.001*
Precipitation (mm)	0	72.20	0.98	3.56	0	0.02	0	0.05*
Relative humidity (%)	6	100	53	21	55	-0.14	-0.003	<0.001*
Sum of ET^1^ (^0^C)	927.7	1555.2	1191.7	162.2	1190.3	0.42	14.73	<0.001*
The beginning of SET^2^ and SEI^3^	5 March	9April	27 March	11.0 days	31 March	0.19	-0.538	0.058
The end of SEI	25 August	17 September	6 September	6.6 days	5 September	0.11	0.176	0.479
The end of SET	7 October	25 November	25 October	10.8 days	25 October	-0.12	-0.333	0.431
The length of SET (days)	184.0	239.0	212.1	13.4	212.5	-0.20	-0.67	0.214
The length of SEI (days)	145.0	187.0	162.5	12.2	161.5	-0.15	-0.4	0.352

1 –effective temperatures (ET); 2 –season of effective temperatures (SET); 3 –season of effective infectiveness of mosquitoes (SEI)

* mark statistically significant values

Trend analysis of thermal conditions for virus replication showed an increase in ET and slight decreases in SET and SEI. Changes were statistically significant for ET only. Statistically significant weakly expressed tendencies in daily temperature increases and relative humidity decreases were also observed. There were no changes in the precipitation rate (Table 5).

**Fig 9:**
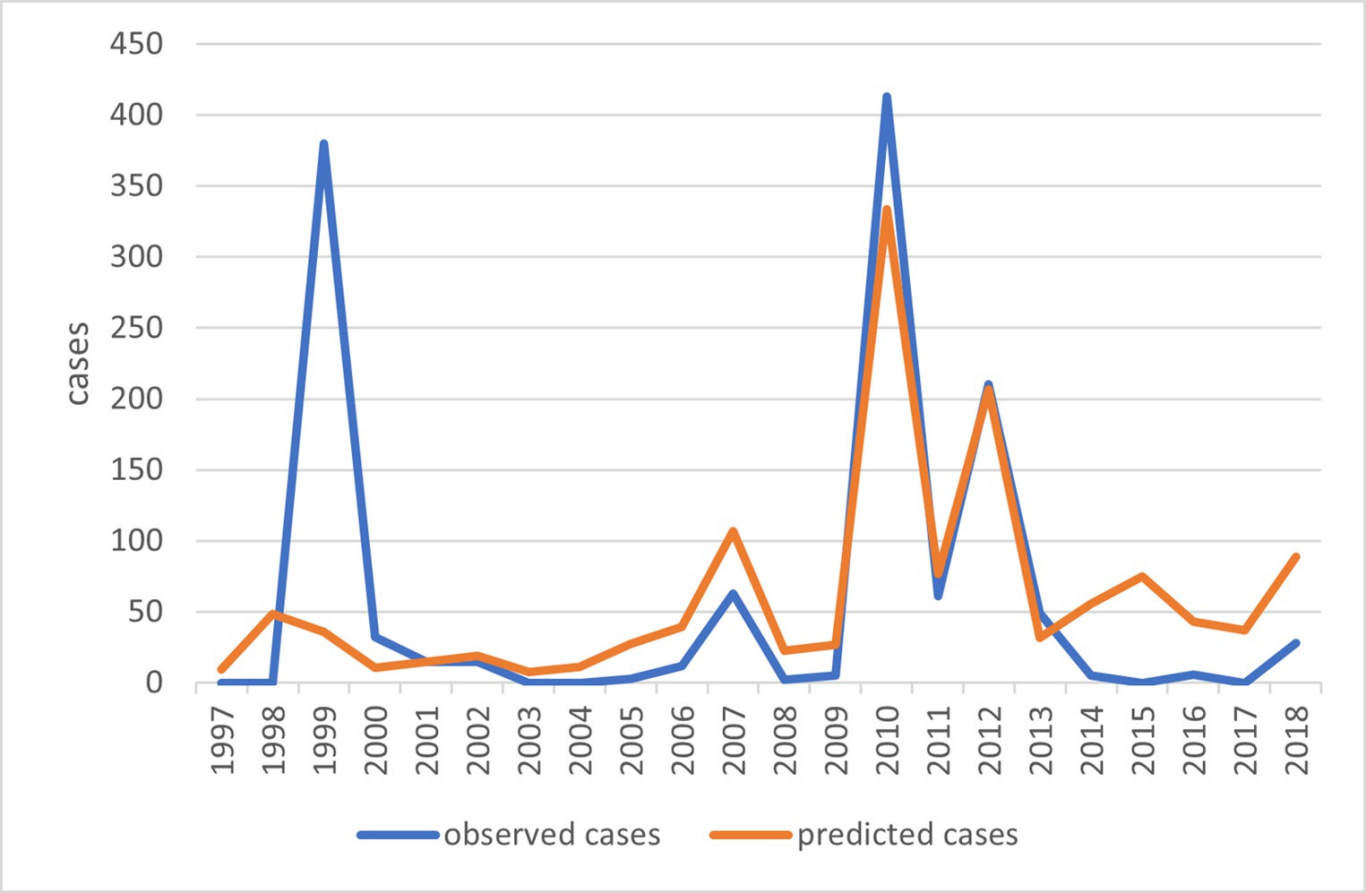
Observed vs predicted number of WNF cases as per negative binomial regression results with the sum of ET as predictor.

A statistically significant correlation between human cases and variables of thermal conditions for WNV transmission was found only for the sum of ET (tau = 0.36, p-value = 0.0224). The regression model showed the statistical significance of the sum of ET to explain yearly variation of cases at p<0.05 level (Fig 9). The distribution of residuals was nearly normal (p-value>0.1 according to Shapiro-Wilk test). SET and SEI varied widely from year to year and there were no differences between outbreak and non-outbreak years.

## Discussion

Since 1999, WNF cases have been reported in Volgograd almost every year, except in 2017. Outbreaks occurred in 1999 (380 cases), 2007 (63 cases), 2010 (521 cases), 2011 (60 cases), 2012 (210 cases), and 2013 (49 cases) [97,98]. Most likely, the number of human infections was much higher than the number of reported cases. A serological survey of the population after the 1999 outbreak revealed that the number of infected humans was 3–10 times higher than the number of laboratory-confirmed cases [99]. Throughout the entire period of WNF case registration in the Volgograd region, the disease prevailed in the urban older age group (over 60), and males had clinical manifestations more often than females [54], which corresponds to the data in other countries. The elderly, immunocompromised, and young individuals were defined as the most affected groups [100–103].

The increase in temperature for the warm period of 1997–2019 was weakly manifested, but affected the growth in the sum of ET required for the replication of the virus (Table 5). Though the 22 year period might be a short time span to consider climate change, our results are in line with other studies that were made for a longer period and indicated a current warming trend in southern Russia [104,105]. At the same time, the durations of the SET and SEI were marginally shorter, although these changes were not statistically significant. These data indicate that Volgograd is a territory that has been consistently favorable for the transmission of the virus.

Platonov et al. [54] demonstrated that outbreaks occurred when mean temperatures in May–July were above 21°С. Recent studies of Shocket et al. [106] observed a peak in incidence at a mean of 24°C in the USA. Our study showed similar results; the 2010 outbreak in Volgograd occurred when the March-October mean temperature was 24°C, which was the maximum observed value for the warm period for 1997–2019, whereas the 1999 outbreak occurred at lower temperatures, with a mean of approximately 20.5°C. The sum of ET was 1188°C during the 1999 outbreak and 1555°C during the 2010 outbreak. The correlation between human cases and the sum of ET allowed us to use ET as an indicator for the possibility of outbreaks. In general, a further increase in temperature may facilitate future outbreaks.

For precipitation, no significant changes in trends were found, while a trend towards a decrease in relative humidity was clearly visible and expected in accordance with the trend towards an increase in temperature. In arid territories, there may be an increase in the incidence after heavy rainfall in some seasons. However, such events may not be reflected in long-term trends. The larvae of the species *Cx*. *modestus* are very sensitive to hydroclimatic conditions. If reeds, as the main breeding sites in Volgograd, are drying, this can lead to a decrease in the population of this species. *Cx*. *pipiens* are more environmentally flexible due to production in man-made sources [107].

The distribution of WNF cases throughout the year is seasonal, with a maximum in August to September in Volgograd [98]. That is, peak clinical disease occurs earlier than the end of the SEI (Table 5). The period of virus replication begins at the end of March (the beginning of SET and SEI) that happens slightly earlier before the massive arrival of migratory birds, including passerine birds, and might indicate local overwintering of the virus. The period of virus replication lasts approximately 5 months. Outbreaks might be facilitated by mild winters, which may contribute to the vector survival [108]. Up to 100%, mortality was observed in *Cx*. *pipiens* populations from Russia, which was associated with average monthly temperatures of -3°C [109]. Date from Southern and Southeastern Europe indicates that temperatures below 2°C and above 6°C have a negative association with WNV infections [110].

Most cases of WNF have been reported in the southern part of Volgograd along major water bodies. In the central part of the city, most cases were concentrated away from the Volga River, such as in green areas along Volga’s small tributaries. Outside the city, WNF cases were aggregated in rural areas, including summer cottage settlements along the Volga River, on the banks of large reservoirs, and near the Volga-Don Canal. Detection of the virus in the environment covered a much larger area, including the Volga-Akhtuba floodplain and islands in the Volga delta.

In addition, the Volga-Akhtuba floodplain can be considered a zone of transfer of virus from the delta areas of the neighbouring Astrakhan region and other territories [111]. The Volga delta is located along one of the main seasonal European-Asiatic avian migration routes (http://www.seen-net.eu), and its wetlands are favorable for nesting [27]. Out of 229 bird species recorded from this area, 227 species migrate to winter localities in Africa, the Indian subcontinent, and the Mediterranean [112] which are WNV-endemic areas. Close contacts between birds during intermediate stops and in wintering places can increase the exposure of birds to vectors, thus facilitating the exchange of pathogens and contribute to the further evolution of the virus.

The leading role of LST and elevation in a model that includes only the ‘natural environment’ and ‘virus detection’ variables confirmed the epidemic significance of the Volga-Akhtuba floodplain. WNV infections were less likely in hot steppe areas (due to the low number of larval mosquito habitats), and more likely at lower elevations of the floodplain. The use of LST as one of the predictors of WNV distribution has been confirmed in other studies [79,113]. A negative correlation with altitude has been found in various areas [41,114,115].

The proximity to water bodies is an important concomitant factor. The likelihood of infection decreases as the distance from water bodies increases. The presence of water bodies and wetlands is especially important in the case of urban-rural virus transmission [116]. However, depending on the type of surface water, the influence can be positive or negative as was found in case of southeast USA [117]. Living in the vicinity of slowly moving water sources was statistically associated with increased risk for human infection, whereas living near moderate moving water systems was associated with decreased odds of human infection. Living near bayous lined with vegetation as opposed to concrete channels also showed increased risk of infection [117]. Similar patterns may be typical for the south of Russia.

Our study showed a gradual movement of the pathogen from the riverine recreational strip to urban areas. A buffer zone might be provided by summer cottage settlements that provide a link between rural and urban transmission. In southern France, it has been demonstrated that WNV foci are associated with heterogeneous agricultural areas [118]. They represent a combination of small agricultural plots separated by natural spaces, which may favor more close contacts of competent vectors and reservoir hosts.

The urban environment could significantly increase the epidemic hazard of the territory by providing favorable conditions for the existence of *Cx*. *pipiens* populations and synanthropic avian hosts. Increased risk due to urbanization has also been found in other regions [55,119]. The link between infection and urban environment can be explained by the fact that in the urban WNV cycle, mosquitoes gravitate towards residential buildings, built-up areas, and areas with moderate vegetation [55,56]. For example, urban microclimates can differ substantially from the macroclimatic background in mosquito habitats [120]. A small number of ditches and detention ponds could be consistent "superproducers" of *Culex* larvae [121]. In southern Quebec and eastern Ontario the importance of the edge of vegetation and mixed or paved areas for the ecology of *Cx*. *pipiens* were highlighted [122].

Suitable larval habitat could be found in basements of buildings if they are flooded and humans were available as a blood meal source. Basement-bred autogenous *Cx*. *p*. *molestus* mosquitoes may play a separate role in maintaining vertical transmission of the virus within mosquito populations, which has been confirmed by many studies [123–125]. However, vertical passage is not efficient for permanent maintenance over many generations without amplification by horizontal transmission, although this might be an additional driver of transmission. In basements, the optimal microclimate for the year-round development of mosquitoes is present [62], thereby maintaining the persistence of the virus during the interepidemic period. Some studies showed that higher temperatures did not increase the transmission rates of biotype *molestus* [35]. The fact that a large number of WNV cases was reported in Volgograd from the area of low-rise dwellings with small houses with garden plots suggests that the role of this vector may be negligible.

Synanthropic bird species, primarily crows and pigeons, are epiornitic amplifiers in urban environments [126,127]. Dead bird occurrences might be correlated with human WNV risk as was observed in some US studies [40,128–130]. However, massive mortality among crows only was observed during the outbreak in Volgograd in 1999 [25,27]. Mortality among the local species *Corvus corone* was not observed in the Astrakhan region despite the widespread involvement of this species in the circulation of the virus, perhaps due to long-term interaction with this virus [25].

In our models, urban areas were determined by the density of roads. Roads identify the places where more people live and road system components such as culverts, storm drains and roadside ditches easily become perfect larval habitat. An increased risk of WNF in areas with higher road density has also been found in Mississippi [130]. The specificity of the Russian road network when not all settlements have a connection with major roads [131] should be considered. Moreover, in urbanized biotopes of Volgograd, the abundance of *Cx*. *modestus* was higher than that in natural environments [31]. The impact of the road density also may indicate that the detection of human cases occurs in frequently visited locations. The results based on virus detection in the environment also suggests that pathogen amplification is efficient in the urban environment.

### Limitation

Unfortunately, the lack of spatial information on avian and mosquito populations in the study area did not allow us to include these variables in the model. Entomological information in Russia is collected extremely unevenly and we do not have qualitative data on the species composition and number of ticks and mosquitoes, as well as vector competence, so we used indirect indicators in this analysis. Another limitation was the lack of environmental data sets with a high spatial and temporal resolution for the study area that could allow for the model to be more accurate. This concerns the improvements in mapping of different types of water bodies and urban landscape heterogeneity using remote sensing data. Limited data availability on the exact locations of infection also complicated the analysis. Moreover, there is lack of full epidemiological investigations for all cases of unexplained fevers.

## Conclusion

The natural features of the Volgograd region formed the conditions for stable long-term circulation of the West Nile virus. At the same time, the urban environment could pose greater hazards for WNV in humans. Quite favorable conditions for the circulation of the virus emerged here, due to both the involvement of competent hosts and vectors and the formation of favorable environmental conditions for their existence. The private building sector with low-storey houses and garden plots located in the city suburban area and Volga large islands provided connections between urban and rural transmission cycles. If case detection is insufficient and diagnosis is not timely in such territories, adverse consequences may develop.

## Supporting information

S1 Fig Climatological information for study area–Volgograd city and its suburbs. (TIF)

S2 Fig Population density map. Contains information from OpenStreetMap and OpenStreetMap Foundation, which is made available under the Open Database License.
(TIF)

S3 Fig LST distribution in Volgograd and its surroundings. Contains information from OpenStreetMap and OpenStreetMap Foundation, which is made available under the Open Database License.
(TIF)

S4 Fig NDWI distribution in Volgograd and its surroundings. Contains information from OpenStreetMap and OpenStreetMap Foundation, which is made available under the Open Database License.
(TIF)

S1 Table Variable contributions according to various spatial models. (DOCX)

S2 Table Model parameterization in MaxEnt software. (DOCX)
