# Supplementary figures and images for: Spatial patterns of West Nile virus distribution in the Volgograd region of Russia, a territory with long-existing foci
Source: PLoS Negl Trop Dis. 2022 Jan 31;16(1):e0010145. doi: 10.1371/journal.pntd.0010145 (PMC8803152; doi:10.1371/journal.pntd.0010145)

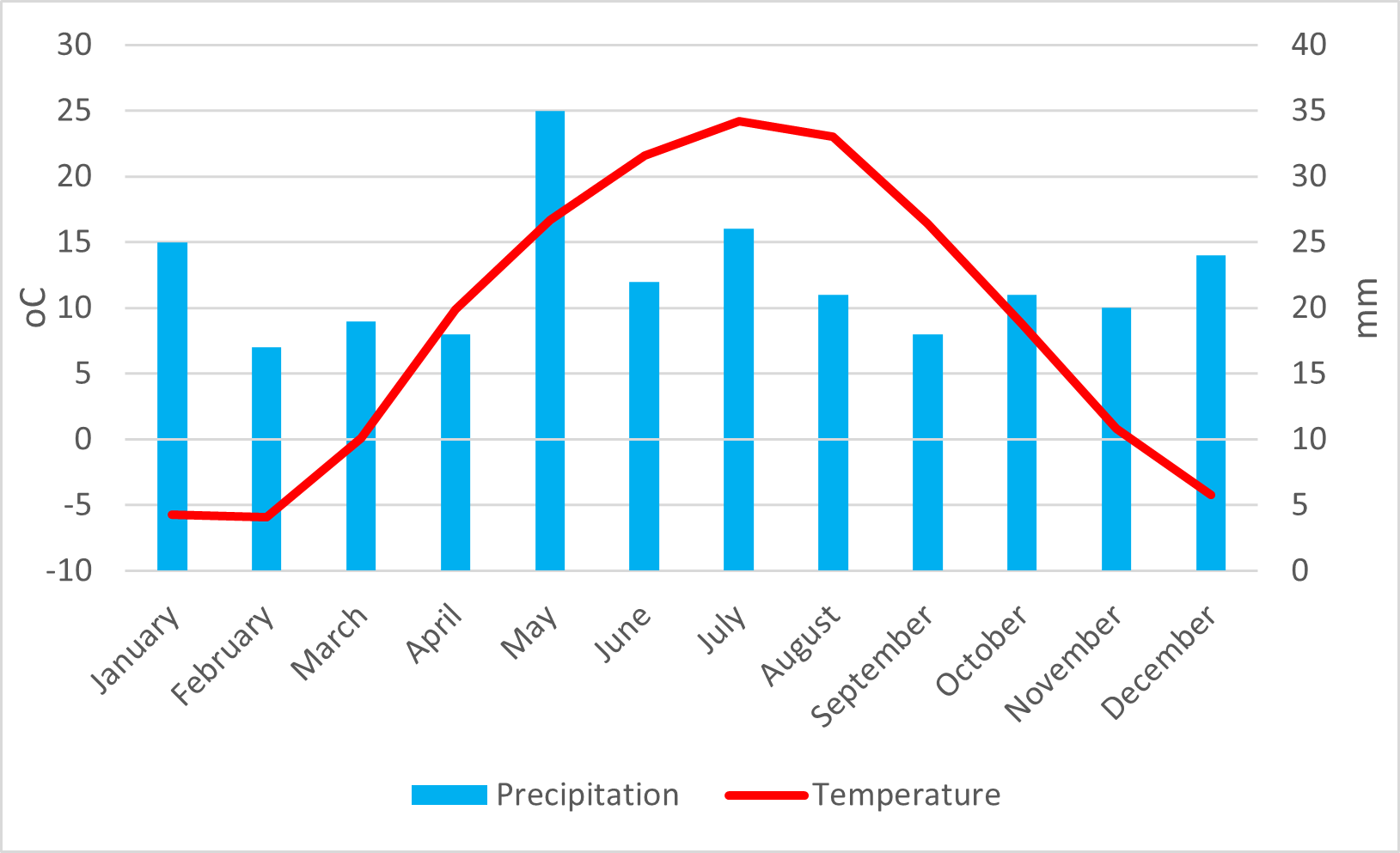

Supplement: S1 Fig — (TIF) [file pntd.0010145.s001.tif]

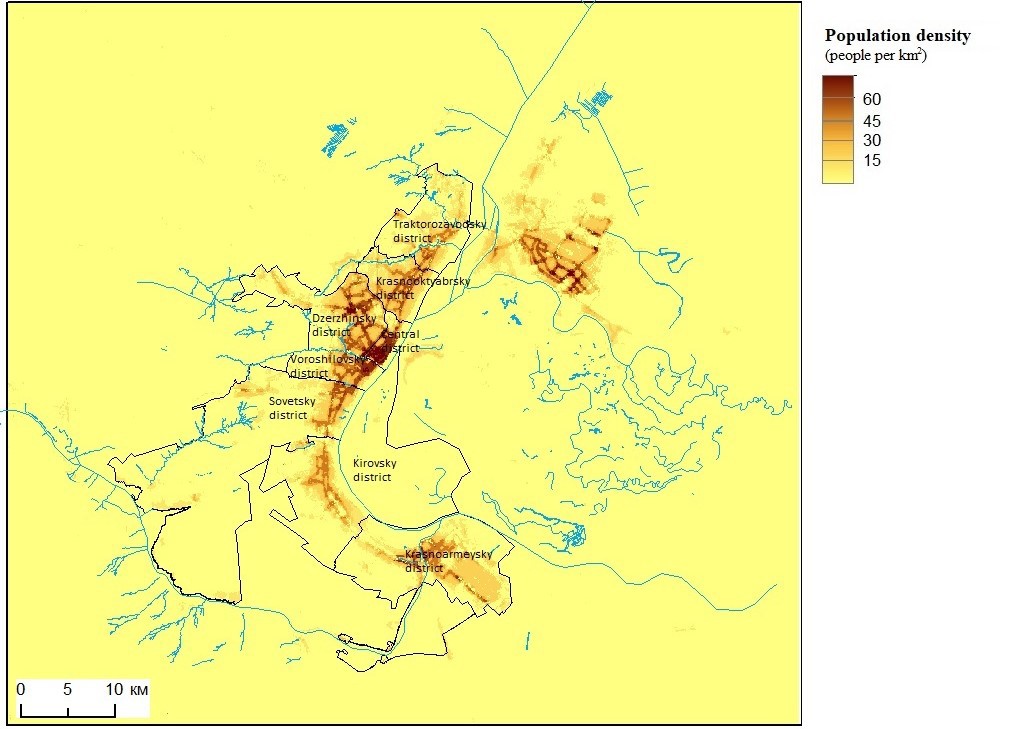

Supplement: S2 Fig — Contains information from OpenStreetMap and OpenStreetMap Foundation, which is made available under the Open Database License. (TIF) [file pntd.0010145.s002.tif]

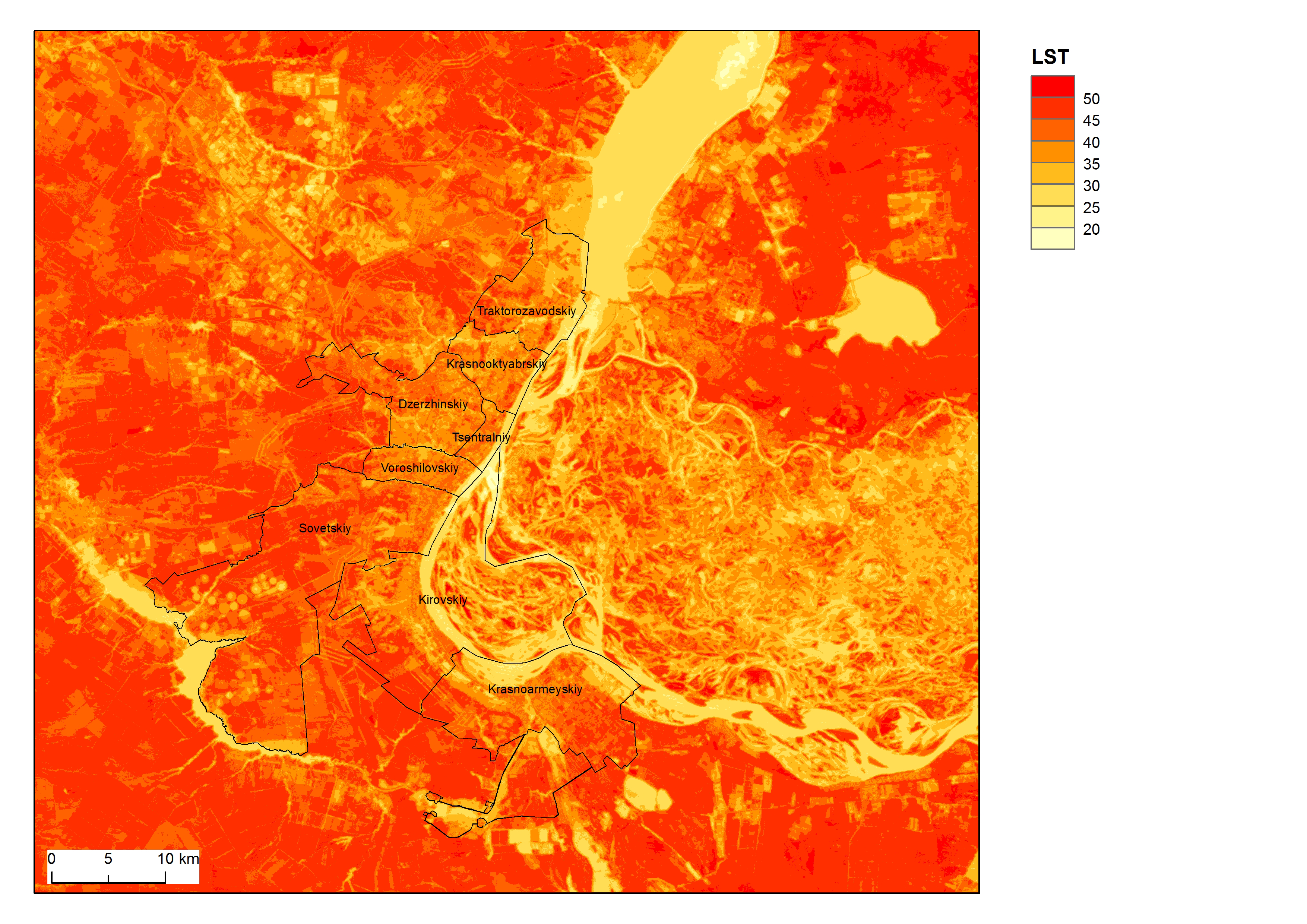

Supplement: S3 Fig — Contains information from OpenStreetMap and OpenStreetMap Foundation, which is made available under the Open Database License. (TIF) [file pntd.0010145.s003.tif]

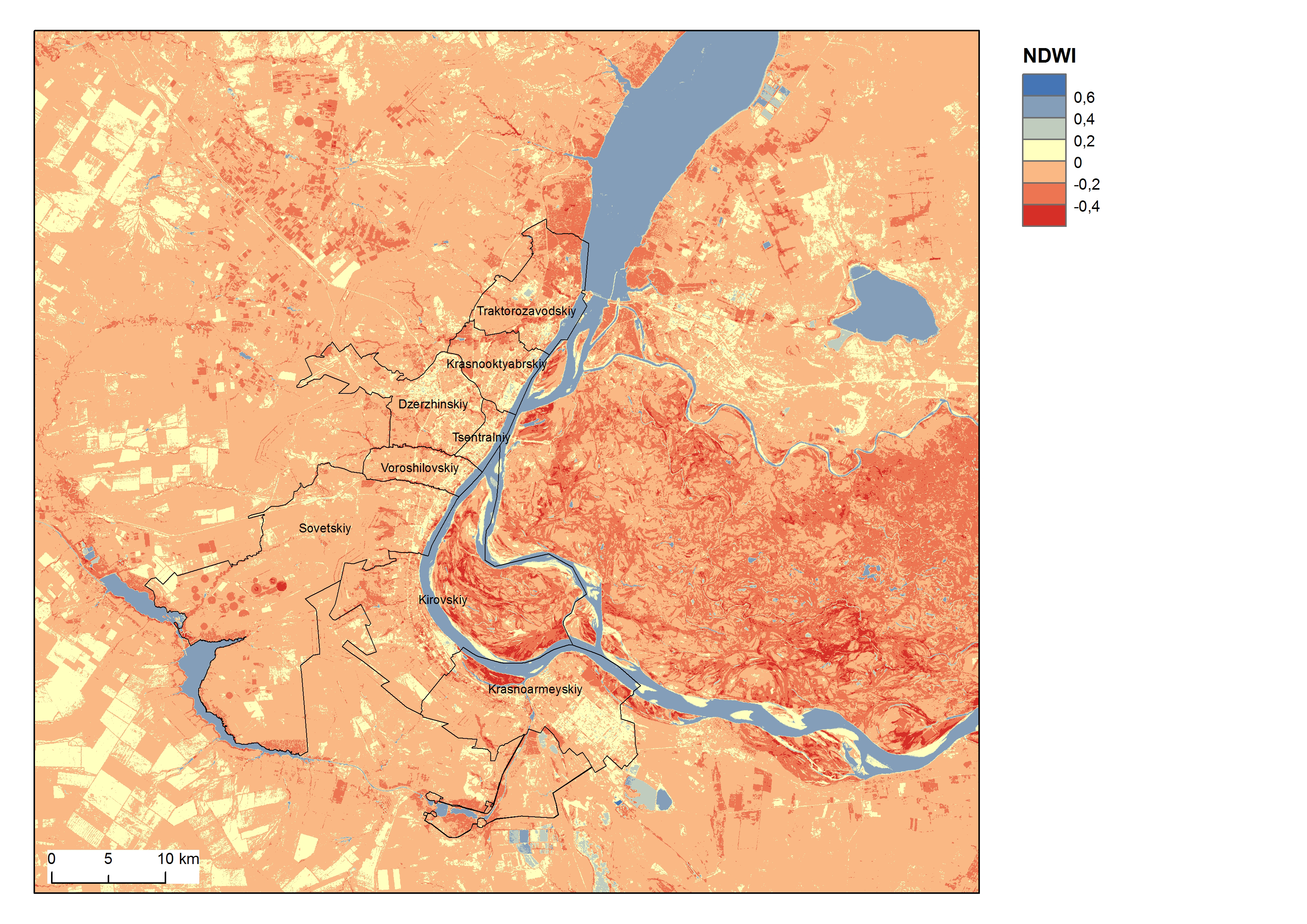

Supplement: S4 Fig — Contains information from OpenStreetMap and OpenStreetMap Foundation, which is made available under the Open Database License. (TIF) [file pntd.0010145.s004.tif]
