# Supplementary material for: Spatial patterns of West Nile virus distribution in the Volgograd region of Russia, a territory with long-existing foci
Source: PLoS Negl Trop Dis. 2022 Jan 31;16(1):e0010145. doi: 10.1371/journal.pntd.0010145 (PMC8803152; doi:10.1371/journal.pntd.0010145)
Supplement: S1 Table — (DOCX) [file pntd.0010145.s005.docx]

**Table 1 Supplementary** Variable contributions according to various spatial models

|  | Model 1  Virus detection sites in the environment as presence data, natural environmental variables | Model 2  Virus detection sites in the environment as presence data, natural and urban environmental variables | Model 1  Possible human infection and virus detection sites in the environment as presence data, natural and urban environmental variables |
| --- | --- | --- | --- |
| Road density | - | 49,6 | 58.6 |
| Building density | - | 12.4 | 6.1 |
| LST | 37.2 | 7.3 | 16.3 |
| Distance to the water bodies | 19.9 | 11.1 | 8 |
| NDVI | 20.7 | 7 | 3.5 |
| Railways density | - | 2.6 | 5.4 |
| Elevation | 22.2 | 9.9 | 2 |
| **Model AUC** | **0.862±0.026** | **0.933±0.015** | **0.948±0.007** |
