# Supplementary material for: Spatial patterns of West Nile virus distribution in the Volgograd region of Russia, a territory with long-existing foci
Source: PLoS Negl Trop Dis. 2022 Jan 31;16(1):e0010145. doi: 10.1371/journal.pntd.0010145 (PMC8803152; doi:10.1371/journal.pntd.0010145)
Supplement: S2 Table — (DOCX) [file pntd.0010145.s006.docx]

**Table 2 Supplementary** Model parameterization in MaxEnt software

| **Parameter** | **Value** |
| --- | --- |
| Random test percentage | 0 |
| Regularization multiplier | 1 |
| Max number of background points | 10000 |
| Replicates | 100 |
| Replicated run type | Bootstrap |
| Maximum iterations | 5000 |
